# Inclusion in Neglected Tropical Disease Programmes: A Review of Inclusive Approaches for Control and Elimination

**DOI:** 10.3390/healthcare14010027

**Published:** 2025-12-22

**Authors:** Ismat Zehra Juma, Opeoluwa Oguntoye, Girija Sankar, Joerg Weber, Babar Qureshi, Juliana Amanyi-Enegela

**Affiliations:** Christian Blind Mission, 64625 Bensheim, Germany; opeoluwa.oguntoye@cbm.org (O.O.); girija.sankar@cbm.org (G.S.); joerg.weber@cbm.org (J.W.); babar.qureshi@cbm.org (B.Q.); juliana.amanyi-enegela@cbm.org (J.A.-E.)

**Keywords:** neglected tropical diseases, NTDs, disability inclusion, elimination, control, gender inclusion, health systems strengthening, DMDI, MMDP, IDM, healthcare

## Abstract

Neglected Tropical Diseases (NTDs) disproportionately affect the world’s most marginalised populations, yet programmes aiming to control and eliminate NTDs often fail to fully address the structural, social, and political dimensions of exclusion. This narrative review examines the concept of inclusion within NTD programming, with a particular focus on intersecting forms of marginalisation, including poverty, gender, disability, and displacement. Drawing on studies from 2010 to 2025, from various databases such as google scholar, PubMed and PLOS, this review synthesises evidence on barriers to equitable healthcare access, the role of community-driven approaches, and the integration of inclusive strategies within NTD programming and broader health systems. Key themes include the impact of structural inequalities such as racism and poverty, the need for gender-responsive services, the marginalisation of displaced communities, and the critical role of community empowerment through mechanisms like peer support and community drug distribution of NTD medicines. The review proposes a working definition of inclusion in NTDs as the intentional integration of underserved groups into all levels of programming, policy, and service delivery. It highlights the urgency of reframing NTDs not just as biomedical challenges but as deeply embedded social justice issues. By embedding inclusion into programme design, implementation, and evaluation, stakeholders can better align NTD responses with global equity goals and the Sustainable Development Goals.

## 1. Introduction

Neglected tropical diseases (NTDs) encompass a diverse group of 21 diseases, including trachoma, onchocerciasis, lymphatic filariasis, soil-transmitted worm infections, and schistosomiasis, among others, that disproportionately affect vulnerable communities in tropical and subtropical regions, inflicting a significant burden on health and well-being [1,2]. Globally, over one billion individuals require prevention or treatment services for at least one NTD, thus accounting for approximately 19 million disability-adjusted life years (DALYs) annually [3].

These conditions have earned the label **“neglected”** because, despite inflicting profound physical and social suffering, they have historically received scant attention in global health. This neglect has manifested across multiple levels: within communities, where fear and stigma discourage diagnosis and care-seeking; within national systems, where health authorities deprioritise remote rural regions lacking NTD support services; and on the global stage, where investments in NTDs are overshadowed by other attention to other infectious diseases [1,2,4,5].

The impact of NTDs extends far beyond acute infection. They are embedded in environmental and socioeconomic deprivation, including inadequate sanitation, poor access to healthcare, and limited educational opportunities, all of which sustain a destructive cycle of poverty and disease transmission [4]. When coupled with ongoing malnutrition, inadequate housing, climate events, conflict, and economic vulnerability, these conditions create fertile ground for NTD epidemics [6].

Additionally, many NTDs result in long-term impairments such as blindness (e.g., trachoma, onchocerciasis), lymphedema (lymphatic filariasis), and physical disfigurement (leprosy). These impairments significantly restrict daily functioning and social participation [7,8]. Compounding these issues, stigma and social exclusion frequently produce adverse mental health outcomes among affected individuals [8,9]. Despite this clear evidence, disability and mental health dimensions remain clearly under-addressed in NTD programmes, which typically emphasise infection control metrics rather than functional outcomes or social inclusion strategies [10].

Recognising this gap, the concept of disability inclusion in NTDs has evolved to encompass broader frameworks for health and social engagement. Rather than merely providing chemotherapeutic medicines, inclusive approaches advocate for equitable access to healthcare and social services, meaningful representation in programme design and decisions, and supportive structures that empower individuals and communities to manage their health and well-being [11]. Hence, this review aims to explore disability inclusion within the context of NTD programmes.

In the context of NTD programming, *inclusion* refers to the intentional integration of historically excluded populations—such as persons with disabilities, women, and displaced people—into all phases of programme design, implementation, and evaluation. This framing is informed by WHO guidance on people-centred health systems and disability equity [12,13]. This framing also aligns with Kuper’s conceptualization of disability inclusion as ensuring that people with disabilities can equitably access and participate in NTD programmes by addressing structural barriers such as physical inaccessibility, financial constraints, stigma, and lack of representation and by involving disability-led organisations in programme planning and delivery [8].

Inclusion is distinct yet interrelated with concepts such as equity and participation:

- **Equity** refers to the fair distribution of resources and outcomes, particularly addressing structural disadvantages [14].
- **Participation** denotes meaningful involvement of affected groups in planning, decision-making, and implementation, but does not always ensure equity or empowerment [15].

Inclusion integrates both dimensions with a particular emphasis on recognising and removing barriers for historically excluded groups. In this review, we look at inclusion as encompassing four interlinked domains, access, participation, empowerment, and governance, each essential for building equitable NTD programmes.

### 1.1. Objective

The primary objective of this review is to explore the multidimensional concept of inclusion in the context of NTDs. Specifically, this review aims to:

- **Define the Concept:** To provide a clear and comprehensive definition of inclusion as it relates to NTDs, considering the various components and dimensions of the concept. Additionally, consider the cross sections of disability inclusion within NTD programming as well as the intersectionality between disability inclusion and NTDs.
- **Explore the Dimensions:** Examine the different dimensions of inclusion, including but not limited to access to healthcare, social participation, gender equity, and their interplay in the context of NTDs.
- **Identify Research Gaps:** Identify gaps in the existing literature, highlighting areas where further research is needed to advance the understanding of inclusion in NTD programmes and policies.

Based on the objectives outlined above, the paper will address the following research question:

How is the concept of inclusion defined and operationalized within NTD programming, and what are the key dimensions and gaps that shape inclusive NTD interventions and policies?

This current paper aims to review existing literature and develop a concept on inclusion within NTDs, specifically focusing on gender and disability.

### 1.2. Significance

Addressing inclusion within NTD programmes is essential for several reasons. First, NTDs are inextricably tied to poverty and inequity, affecting over one billion people living in 149 countries, making inclusion a matter of social justice and human rights [12,16,17]. Second, inclusive NTD services can be contextual, community-driven, and sustainable. Recent estimates indicate that around 1.62 billion people required NTD interventions in 2022, a 26% decrease since 2010, highlighting how tailored programme strategies can successfully increase treatment uptake [18,19]. Additionally, inclusion aligns with broader global health and development agendas, including the Sustainable Development Goals (SDGs), which emphasise the importance of leaving no one behind [20].

On the other hand, approximately 15% of the global population has a disability [21,22,23], and this large group must be considered when designing NTD programmes. Improving the inclusion of people with disabilities may require adaptations to NTD programmes, such as making them physically accessible or training staff about disability awareness. Without incorporating disability within NTD programmes, the quality of life of people with NTDs will suffer, and global targets for elimination and management of NTDs will not be met [7].

This review fills a significant gap in the existing literature by consolidating current knowledge and conceptual frameworks in relation to inclusion in NTDs. Synthesising evidence from diverse contexts and disease-specific studies provides policymakers, researchers, and practitioners with a resource for designing more inclusive and practical strategies to combat NTDs. Ultimately, this review contributes to the broader discourse on global health equity and underscores the imperative of inclusion as a central tenet in the fight against NTDs.

## 2. Methodology

This review employed a narrative synthesis, allowing for an in-depth and interpretive examination of the literature on disability inclusion in neglected tropical disease (NTD) programming. Compared to systematic reviews, narrative reviews offer flexibility that accommodates diverse study designs and methodologies, allowing nuanced thematic exploration [24].

### 2.1. Search Strategy

A comprehensive search was conducted across PubMed, Scopus, and Web of Science, covering the period from January 2010 to April 2025. Medical Subject Headings (MeSH) and free-text search strings, including terms such as “*neglected tropical diseases*”, “*disability inclusion*”, “*equity*”, “*community participation*”, “*stigma*”, and “*rehabilitation*” were used. These were combined using Boolean operators (AND/OR), and search terms were iteratively refined according to guidelines by Sukhera [24].

### 2.2. Screening and Eligibility

**Figure 1:**
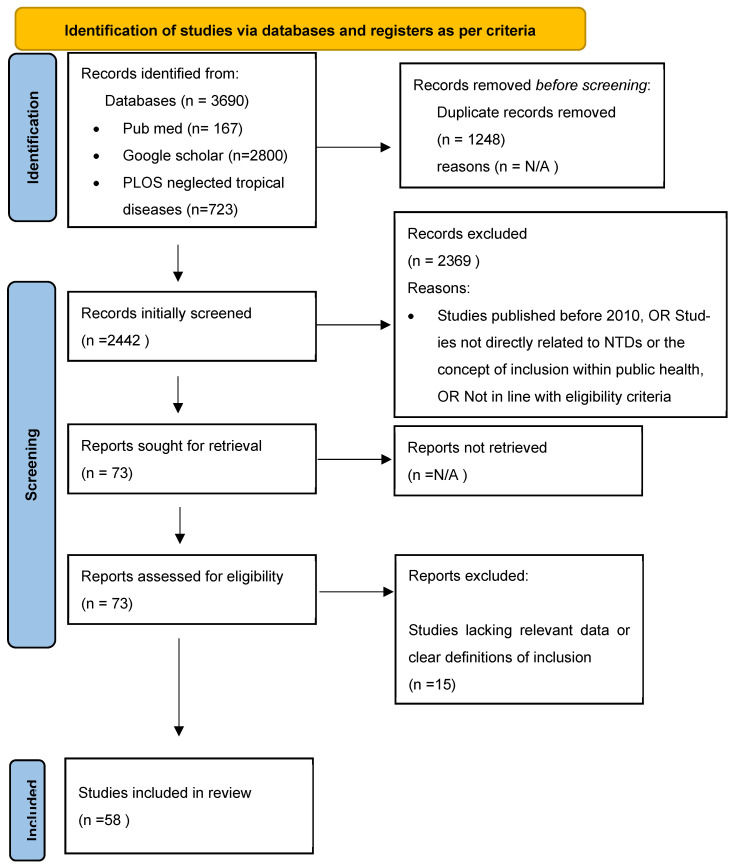
PRISMA Flow Chart.

We followed the SALSA framework (Search, Appraisal, Synthesis, Analysis) to guide selection [25]. Two reviewers conducted an initial screening of titles and abstracts independently, followed by a full-text assessment. Eligibility criteria, as shown in Table 1, included: (1) English-language publications from 2010 to 2025 focusing on disability inclusion within NTD programming; (2) primary empirical studies (quantitative, qualitative, mixed methods), programme evaluations, or policy analyses explicitly addressing disability adaptations, data systems, or community participation; (3) policy or grey literature with relevant insights. Exclusion criteria encompassed laboratory-only articles, clinical trials without disability focus, grey literature lacking sufficient methodological detail, and non-NTD studies. Figure 1 shows the PRISMA flow for screening and selection of articles.

### 2.3. Data Extraction and Synthesis

From included studies, we extracted data on how disability inclusion was defined, integrated into NTD service delivery, and evaluated; types of programme adaptations; reported outcomes; enabling conditions; and implementation obstacles. Dual independent screening and data extraction were used, with disagreements resolved through discussion. Although we did not apply a formal risk-of-bias tool—consistent with narrative review conventions—we conducted an informal critical appraisal based on the Mixed Methods Appraisal Tool (MMAT), focusing on methods clarity and contextual framing.

Thematic content analysis was performed iteratively, with emerging themes organised around our three review questions: conceptualization, practical strategies, and barriers to inclusive implementation.

### 2.4. Ethical Considerations

This review was based entirely on secondary information, which is publicly available literature and did not involve primary data collection; hence, no formal ethical approval was required [24]. Nonetheless, ethical standards were upheld by ensuring the accurate representation of prior research, giving proper credit to original authors, avoiding any distortion of their findings, and selecting sources based on their relevance, credibility, and methodological quality [26]. Sources were selected based on relevance, credibility, and methodological rigour to ensure the integrity of the synthesis [27].

## 3. Results and Findings

This narrative review included studies comprising primary research articles and systematic reviews published between 2010 and 2025 that met our inclusion criteria. The selected literature encompassed qualitative, quantitative, and mixed methods designs, with a predominant focus on sub-Saharan Africa (Nigeria, Uganda, Kenya, Cameroon, Ethiopia, and Liberia) and Latin America (Brazil). Several studies also addressed transnational and global health policy contexts related to disability inclusion in NTD programming. Drawing from this rich corpus, we distilled key thematic insights, clarified emerging concepts, and identified critical research gaps. It is essential to note that, although comprehensive in scope, this review does not exhaustively cover disability inclusion theories; thus, it limits its focus to studies most relevant to our research questions.

As shown in Table 2, the synthesis of the eligible studies highlights five overarching themes, each examined through specific methodologies, in targeted geographies, and with distinct populations, offering essential insights into the current landscape of disability inclusion within NTD programmes.

Below, each theme is explored in-depth based on the findings of the review.

### 3.1. Social and Economic Inequalities in NTD Programmes

A substantial body of evidence underscores how social and economic determinants profoundly influence both the burden of NTDs and individuals’ ability to access care. Factors such as poverty, geographic marginalisation, displacement, systemic racism, and inadequate infrastructure consistently emerge as barriers to equitable treatment in endemic regions [28,29,30,31]. People with disabilities are disproportionately impacted by systemic poverty and social exclusion, especially when combined with low-income or rural settings. Disability often intersects with racial and economic marginalisation, leading to multi-layered vulnerabilities [28]. While gender itself is a pivotal axis of social exclusion, it has been intentionally examined as a distinct theme in this review to fully highlight its unique and intersecting impacts on disability and inclusion within NTD programming.

#### 3.1.1. Racism

Research in Brazil has revealed a clear link between systemic racism and the disproportionate impact of NTDs on black and marginalised communities [28]. In Brazil, Black Brazilians with disabilities are largely invisible in health systems due to a lack of disaggregated data, making it difficult to ensure they benefit from NTD programmes [28]. Historical and structural inequities have limited access to healthcare, education, and economic opportunities, exacerbating disease burden among these groups. One study emphasises that such communities are routinely denied “decent living conditions, health, and social justice stemming from institutional racism,” a dynamic that reinforces their invisibility in health systems and research agendas [28].

The authors further argue that insufficient investment in research and development for NTD prevention and treatment deepens this inequity, underscoring the need for inclusive public health approaches that address both medical and social determinants of health. Additionally, black individuals with disabilities in Brazil remain largely invisible in health systems due to a lack of disaggregated data by race and disability, making it difficult to assess their healthcare engagement or advocate for targeted interventions [32]. This invisibility perpetuates inequities and policy neglect. Moreover, systemic intersectional discrimination severely increases their vulnerability: they are overrepresented among the homeless, institutionalised, and incarcerated populations, and are disproportionately exposed to violence, including police brutality and gender-based abuse [33].

#### 3.1.2. Poverty

Poverty is a fundamental driver of both the persistence and spread of NTDs, disproportionately impacting populations with limited financial resources, inadequate access to healthcare, and scarce prevention and treatment options [28,34]. This vulnerability is compounded by socioeconomic factors such as gender inequality, unemployment, illiteracy, malnutrition, political instability, and poor sanitation and education systems, which collectively increase exposure risk and constrain the effectiveness of disease control strategies like mass drug administration (MDA) [28,35].

In Brazil, economically disadvantaged regions bear the highest NTD burdens, including those caused by schistosomiasis, leprosy, and chagas disease, highlighting the necessity of health interventions that go beyond biomedical solutions and emphasise social justice, improved living conditions, and inclusive health frameworks [3,28]. Across marginalised communities, lack of essential services like clean water and sanitation further impedes NTD prevention, underscoring the urgent need for universal health coverage and equitable access to treatment [3,28].

Research further demonstrates the strong correlation between socioeconomic status and the prevalence of NTDs. A systematic review analysing over 5500 studies indicated that lower socioeconomic status correlates closely with elevated risk of NTDs, some twice as high, illustrating a clear social gradient in disease prevalence [36]. This inequality is particularly evident in diseases such as soil-transmitted helminth infections (STH), visceral leishmaniasis (VL), and schistosomiasis. While wealthier individuals in endemic areas may also be affected, the broader trend reflects a social gradient in disease burden, underscoring the urgent need for equity-focused interventions in NTD surveillance and monitoring systems [36]. To address this, equity-focused interventions must integrate socioeconomic indicators into surveillance systems, ensuring the most vulnerable are reached.

Even though MDA remains central to NTD control, significant gaps persist in treatment access and surveillance, particularly among marginalised groups. Individuals excluded from these programmes often remain invisible in the data, artificially inflating reported programme success. Modelling studies, such as those by Clark et al. [34], reveal that overlooking these excluded groups diminishes the likelihood of achieving elimination goals. This disconnect between treatment and surveillance data highlights the crucial need to engage these populations to ensure accurate monitoring, effective evaluation, and ultimately, the elimination of NTDs [29,33].

#### 3.1.3. Displacement

Displacement presents substantial and intersecting challenges to embedding disability-inclusive approaches within NTD programmes. Roughly one in six forcibly displaced people worldwide lives with a disability, yet they commonly encounter inaccessible facilities, shortages of assistive devices, a dearth of sensory-adapted information, and exclusion from registration databases, factors that collectively restrict their access to NTD services in camps and other humanitarian settings [37,38]. Furthermore, disability prevalence is higher among the forcibly displaced and economically disadvantaged populations, yet they often remain excluded from NTD surveillance and drug distribution systems due to inaccessibility and stigma [39,40]. The Ascend programme in Niger demonstrates how these gaps can be narrowed by linking UNHCR registration with disability identifiers, overlaying treatment maps with camp-level inclusion data, and recruiting caregivers with lived experience as community drug distributors who use context-appropriate communication modes. As a result, the Ascend programme has shown that equitable coverage is achievable [41]. Nevertheless, such practices remain exceptions; most routine operations in fragile settings continue to omit persons with disabilities because of structural inaccessibility and limited planning [42].

Intersectional analysis further reveals that disability, displacement, gender, and poverty compound marginalisation. UNHCR’s Working with Persons with Disabilities in Forced Displacement toolkit urges agencies to capture disaggregated data, guarantee accessible emergency messaging, and involve persons with disabilities in decision-making, yet these recommendations are rarely implemented in NTD campaigns [37,43]. Displaced people with disabilities are also at heightened risk of violence, homelessness, and service loss from theft of wheelchairs to gaps in psychosocial support undermining trust in health providers and reducing treatment uptake [44].

Delivering disability-inclusive NTD programmes hinges on four mutually reinforcing measures: first, ensuring that both UNHCR and national NTD databases systematically capture disability status using tools such as the Washington Group questions to make people with disabilities visible in planning and resource allocation [41,45]. Additionally, redesigning service delivery so that treatment sites are accessible, and informational materials are designed to meet diverse physical, sensory and communication needs [46]; furthermore, meaningfully engaging displaced persons with disabilities by training them to serve as peer educators and community drug distributors, thereby increasing trust and contextual relevance [47]. Incorporating disability-sensitive indicators into routine monitoring so coverage gaps and access barriers can be identified and addressed through adaptive management is also critical [12,39]. Without this integrated approach, even programmes that incorporate a “leave no one behind” approach would risk systematically overlooking displaced persons with disabilities, deepening health inequities and undermining global NTD-elimination targets [48,49].

### 3.2. Gender Equity in NTD Programmes

Gender remains a critical yet persistently undervalued determinant of success in NTD control and elimination. Biological sex alone does not explain differential risk; rather, exposure, treatment access and long-term disability are mediated by intersecting inequities such as poverty, geographic isolation and disability status [50]. Programmes that fail to integrate gender analysis run the risk of reinforcing these power imbalances, under-serving large segments of the population and, ultimately, missing elimination targets [50].

Evidence from 16 African and Asian countries shows that coverage data disaggregated by gender routinely uncover hidden gaps; in 3% of districts male uptake exceeded female uptake by ≥10 percentage points, signalling structural barriers for women and girls [51]. Uganda’s National NTD Control Programme illustrates these barriers in practice: men were more likely to miss treatment because of labour migration, whereas community drug distributors lacked guidance on treating pregnant or breastfeeding women, leading to systematic under-treatment of this group [52]. In Ulanga, Tanzania, coverage gaps in mass drug administration were exacerbated by gender-related dynamics, including women’s higher participation in ivermectin distribution compared to men, and greater community trust in female distributors. However, structural issues such as poor timing of campaigns during farming seasons and absence of tailored guidance for gender-sensitive delivery continued to affect overall coverage [53].

While gender-informed approaches may increase short-term costs, they ultimately lead to more sustainable and inclusive outcomes, enabling NTD programmes to contribute meaningfully to the goal of universal health coverage without reinforcing existing inequalities [50]. Gender-responsive frameworks, such as those proposed by Theobald and colleagues, advocate three core actions: (i) sex and age-disaggregated monitoring; (ii) community dialogues that address household power dynamics; and (iii) explicit budgeting for gender-sensitive delivery (e.g., women CDDs, pregnancy-safe guidelines) [50]. Where implemented, these measures have yielded measurable gains. For example, programmes that recruited women as CDDs in Malawi and Nigeria reported higher trust among female beneficiaries and increases of 6 to 12 percentage points in female coverage [54].

Importantly, gender intersects with disability, and women with disabilities face amplified stigma, mobility constraints and heightened exposure to gender-based violence, all of which deter health-seeking and limit participation in NTD campaigns [55]. Yet disability-sex disaggregation is virtually absent from routine surveillance, obscuring inequities and hampering corrective action [4].

**Figure 2:**
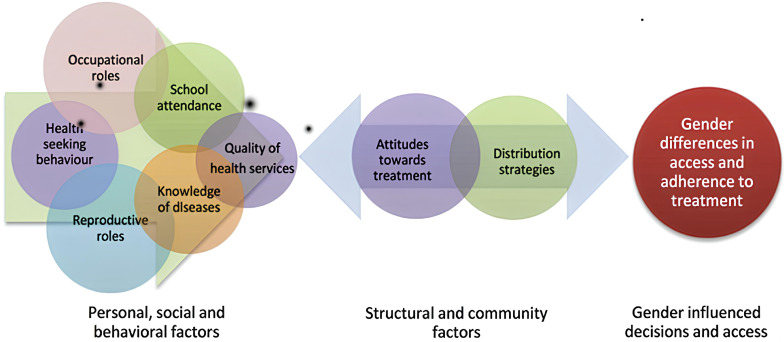
Framework for gendered differences in access and adherence to NTD treatment programmes [52].

Expanding on existing frameworks, Figure 2 in the study highlights the central role of community attitudes and distribution strategies, illustrating that a more comprehensive understanding of the complexities of community-based treatment programmes is essential. Gender-related obstacles, such as social norms and unequal power dynamics, must be addressed to ensure the future success of these programmes, particularly in contexts where community attitudes and distribution methods play a pivotal role in treatment access and adherence [52].

Gender exerts a decisive influence on how people experience and seek care for NTDs in Ethiopia. Qualitative work across five endemic diseases including lymphatic filariasis, podoconiosis, schistosomiasis, soil-transmitted helminth infections and trachoma shows that restrictive social norms, unequal household power and stigma surrounding genitourinary symptoms delay or prevent women from presenting for treatment, problems compounded by low community awareness and discomfort discussing “private” complaints [56]. Wharton-Smith and colleagues therefore argue that national NTD strategies must move beyond binary sex categories and adopt intersectional gender analyses that consider disability, poverty, and geography to achieve universal coverage [56].

**Figure 3:**
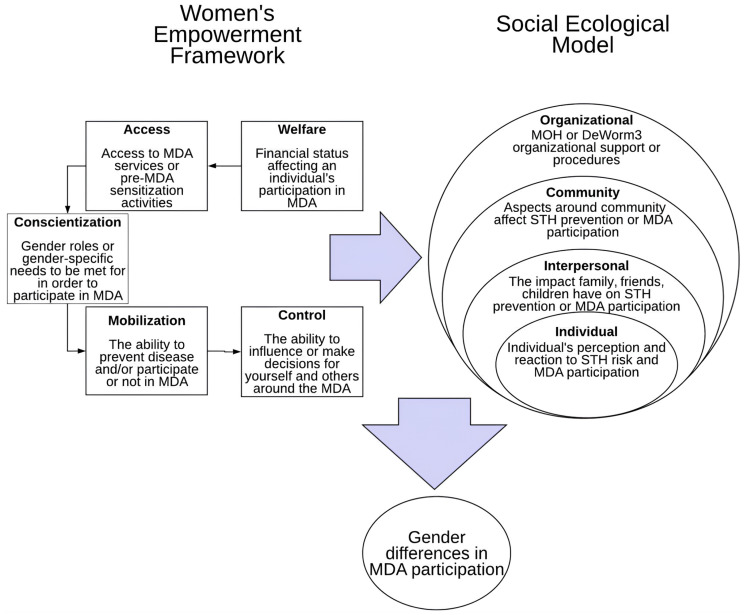
Welfare, Access, Conscientization, Mobilization, and Control (WEF) framework and the Social-Ecological Model (SEM) [57].

Comparable patterns emerge in West Africa. In Comé, Benin, focus-group research revealed that both men and women valued the economic benefits of soil-transmitted helminth MDA. Yet, women were sceptical after previously disorganised campaigns and demanded a voice in planning, safer timing, and door-to-door delivery to increase control over household decision-making [52,57,58]. Men, by contrast, downplayed their risk of infection and were less concerned about the delivery mode [57]. Applying the Welfare, Access, Conscientisation, Mobilisation, and Control (WEF) framework, together with the Social-Ecological Model (SEM), shown in Figure 3, Geyer et al. demonstrated that gender-tailored MDA, mainly when led by female community drug distributors (CDDs), improved coverage and equity at every societal level [57].

These findings echo cross-country evidence from Ethiopia and Tanzania, where routine NTD surveillance systems lacked sex- and disability-disaggregated indicators, making women with disabilities nearly invisible in programme planning. The study also highlighted critical gaps in pregnancy-specific treatment guidance and emphasised that meaningful involvement of women with disabilities in programme design is essential for inclusive NTD interventions [51]. While gender-responsive approaches may incur initial costs, they lay the foundation for more equitable and sustainable progress toward universal health coverage [51].

Ultimately, gender equity should be a central focus in NTD programmes. This includes allocating time for community sensitization and developing educational messaging that addresses women’s specific concerns, especially regarding fertility and treatment safety. Collaborative, multisectoral approaches, especially those involving reproductive and maternal health professionals, are crucial for addressing NTDs such as female genital schistosomiasis. Achieving the Sustainable Development Goals (SDGs), which focus on reducing health disparities and expanding access to healthcare, will significantly contribute to the elimination of NTDs. Progress in these areas will depend on strong, coordinated efforts from governments, civil society, researchers, funders, and communities [59].

### 3.3. Inclusion in Intervention Delivery and Health System Integration

Inclusive delivery and strong health-system integration remain the decisive bottlenecks for taking proven NTD treatments to the people who need them most. Evidence from multiple settings indicates that weak primary-care platforms, shortages of trained staff, and sporadic community engagement systematically undermine the impact of MDA campaigns and case-management services [60,61]. Scholars therefore emphasise inter-sectoral collaboration, enforceable reporting rules, and genuinely “bottom-up” approaches that give communities power to diagnose their own priorities and co-create solutions [62].

In western Kenya, Ochola, Karanja, and Elliott utilised Sen’s capability lens to demonstrate that when villages define success in their own terms, such as determining safe water points, locally chosen drug distribution sites, and establishing transparent feedback loops, MDA coverage rises, and social trust deepens [60]. Their findings underline that national strategies will only be sustainable when local capability and national priorities are aligned.

Nigeria’s Equity-PAR project demonstrates how this can be implemented at scale. Piotrowski et al. [61] identified nine iterative steps, from participatory mapping and cross-sector working groups to routine “learning health-system” reviews, that increased ivermectin coverage in two states and embedded continuous reflection into district plans [61]. Similar participatory micro-planning tools have since been piloted in Ghana and Sierra Leone with promising early results [48].

Yet significant gaps persist. Rigid distribution timetables, myths about infertility, and the absence of pregnancy-safe guidelines all decreased uptake, while school-based models missed pupils in private, informal, or Quranic schools [62]. Modelled data from Mali, meanwhile, show that leaving “never-treated” mobile households out of surveillance reduces the likelihood of reaching elimination thresholds by up to 30% [48].

Liberia’s Disease Management, Disability, and Inclusion (DMDI) platform demonstrates the potential of health-system integration, as it links case management with rehabilitation and psychosocial services. As a result, Liberia cut the average travel time in half and improved treatment completion by a quarter [62]. WHO now encourages such cross-programme collaboration as part of its 2030 road map targets [63].

CDDs, often women, remain the “foot-soldiers” of MDA. Where they receive disability and gender training, trust rises and coverage gaps narrow; where they do not, fear and misinformation flourish [64]. Mobile migrant reviews confirm that mixed-gender CDD teams are more successful in reaching herders, artisanal miners, and displaced households than male-only teams [40,42].

Collectively, these studies demonstrate that re-centring equity necessitates three reinforcing steps: empowering communities through participatory planning, integrating NTD services with broader, people-centred health functions, and monitoring who remains missing using sex-, age-, and disability-disaggregated metrics. Without this triad, programmes risk perpetuating the very inequities they seek to solve. Recent WHO guidance calls for programmes to collect and act on disability-disaggregated data, yet most countries lack such metrics, making it harder to identify exclusion patterns [12].

### 3.4. Inclusion in Health Systems to Combat NTDs

NTD programmes are shifting from vertical, drug-centric campaigns toward integrated, people-centred delivery models that can be sustained by national systems. Liberia’s Disease Management, Disability, and Inclusion (DMDI) platform illustrates this evolution: the strategy bundles case management, rehabilitation, and psychosocial services for five skin-NTDs and is now embedded in county health plans, although donor dependence and fiscal shortfalls still threaten scale-up [62]. Experience elsewhere echoes Liberia’s lesson. A systematic scoping review of found that integrating NTD activities into primary health services improves coverage and cost-effectiveness, provided there is strong governance, stable financing, and active community participation [65]. Yet many health systems continue to overlook equity: women, children, and persons with disabilities are under-represented in registers and often absent from decision-making bodies. Intersectional, gender-sensitive planning, as recommended by global analyses that frame NTDs as a “litmus test” for universal health coverage, must therefore be embedded from the outset [13].

Recent evidence from Nigeria confirms the value of participatory action research: nine iterative steps, ranging from community mapping to inter-ministerial working groups, have improved ivermectin coverage and normalised continuous learning across sectors [61]. Similar gaps have been documented among mobile agricultural workers in Mali and artisanal miners in Ghana, where failure to adapt MDA schedules to labour patterns reduced projected elimination probabilities by up to 30% [63]. International funding volatility compounds these risks: recent cuts to a significant bilateral NTD portfolio may leave 100 million people without treatment and strand nearly US $1 billion in donated medicines [66].

To make integrated care durable, countries must align with the WHO 2030 road-map pillars, change operating models and culture to facilitate country ownership, which highlights people-centred delivery, cross-programme collaboration, and country ownership [1,67] while mobilising domestic resources to reduce donor dependence [68]. Emerging models place local civil-society organisations at the centre of micro planning and accountability, arguing that “last-mile” equity hinges on grassroots legitimacy and social capital [68]. Sustainability research further emphasises the need for explicit metrics that capture equity, financing, and community empowerment, in addition to disease prevalence [66].

### 3.5. Inclusion of Community Members in NTD Programmes

NTD programmes have long relied on community mobilisation, yet many still focus narrowly on biomedical endpoints, overlooking the psychosocial realities and disability-specific barriers that shape uptake and long-term well-being [69,70]. High levels of depression and anxiety were found among people affected by skin NTDs, especially women, with stigma and disability as key predictors. Community-based responses, including peer support, are urgently needed to reduce psychological distress and improve treatment outcomes [71,72]. When such groups are co-designed with individuals with disabilities, they generate accessible meeting formats, tactile or pictorial health education, and caregiver accompaniment, thereby increasing participation among people with visual, hearing, or mobility impairments [70].

Community-directed distributors (CDDs) remain pivotal for last-mile service delivery, having treated more than about 200 million people annually across 27 countries; yet disability training is rarely part of their curriculum [64]. Studies from countries such as Cameroon and Uganda reveal that CDDs often exclude pregnant women, persons with mobility limitations, and the deaf community, mainly because they lack guidance on pregnancy-safe regimens or sign-language skills [58,73]. Inclusive refresher training covering accessible communication, stigma reduction, and safe drug protocols for pregnant and breastfeeding women has raised female and disability-specific coverage by 8 to 12 percentage points in pilot districts [58,73].

Systematic reviews indicate that CDD motivation is contingent upon social recognition and modest financial or in-kind incentives; when these incentives are absent, attrition rates increase and coverage among hard-to-reach groups declines [74]. Policy briefs now recommend formalising CDD roles within national community-health strategies and integrating disability indicators into routine supervision tools, aligning with the WHO’s 2030 Road Map commitment to people-centred care [1].

Ultimately, inclusive community engagement must combine three elements: (i) peer support groups that tackle psychosocial and livelihood needs; (ii) disability-trained, gender-balanced CDD cadres; and (iii) monitoring systems that disaggregate data by sex, age, and disability. Without these, programmes risk perpetuating the very exclusions that drive NTD transmission and disability in the first place [74,75,76].

## 4. Discussion

NTD control cannot be judged a success until programmes reach and empower the communities that carry the heaviest burden. Evidence synthesised in this review shows that inclusion is not a “value-add” but a pre-condition for effective, equitable and sustainable NTD elimination. Yet blind spots remain pervasive: the poorest rural households, displaced communities, women, and persons with disabilities continue to fall through the cracks of surveillance, drug distribution, and policy design.

For example, in their study, Clark et al. show that untreated and unobserved groups (those excluded from drug distribution) also tend to be excluded from surveillance, leading to bias in what is observed vs. what is happening in the full eligible population, impacting the implementation of NTD programmes as well as their evaluation [34].

Systemic poverty, gender inequality, racial discrimination, and forced displacement continue to shape who is infected, who receives care, and who is left behind. Importantly, people with disabilities represent a sizeable and routinely invisible segment of every one of those vulnerable groups.

Our synthesis demonstrates that social and economic inequalities remain deeply embedded in the persistence and spread of NTDs. The disproportionate burden borne by Black communities in Brazil and marginalised groups in sub-Saharan Africa illustrates how systemic racism and poverty intersect to deepen exclusion from healthcare access and surveillance systems [28,59]. These findings align with Houweling et al.’s [36] review, which showed that NTD prevalence strongly correlates with low socioeconomic status, making equity-focused interventions an imperative rather than a luxury.

Gender emerged as another critical but under-addressed axis of exclusion. Multiple studies highlighted the gendered barriers to NTD treatment access, including power imbalances, lack of sex-disaggregated data, and inadequate training for treating pregnant or lactating women [50,52]. These gaps point to the urgent need for intersectional and gender-responsive strategies that address not only health access but also broader social determinants of health, such as decision-making authority and caregiving roles [56,57].

The review also underscores that displacement due to conflict, climate change, or economic instability exacerbates invisibility in NTD programming. As Harvey et al. [41] discuss, displaced populations often fall through the cracks of national health systems, highlighting the need for multisectoral collaboration and data innovation to fully implement the “Leave No One Behind” (LNOB) principle in practice.

Encouragingly, several studies demonstrate how inclusive intervention models can help bridge these divides. Participatory action research (PAR) approaches and community-driven planning processes, such as those trialled in Nigeria and Kenya, show promise in realigning programmatic priorities with local needs [60,61]. These approaches not only increase treatment uptake but also foster trust and long-term engagement, crucial for the sustainability of NTD efforts.

The role of community-led initiatives, particularly peer support groups and CDDs, emerged as vital for delivering integrated and person-centred care. While such models are cost-effective and highly adaptable, they remain undervalued within national health systems, often lacking proper incentives, training, or recognition [71,75]. Systemic support for these grassroots actors is key to realising inclusive health outcomes and building resilience against future public health threats.

Finally, the integration of inclusion into health systems rather than isolated programmes is a consistent recommendation across the literature. Liberia’s DMDI model [62] and systematic evidence from Branda et al. [3] emphasise that mainstreaming disability, equity, and gender considerations into national health policies leads to more efficient and impactful outcomes. However, barriers such as fragmented funding and weak governance structures continue to hinder progress.

Based on the analysis above, we would also like to put forth a definition of inclusion within NTDs:

Inclusion in NTDs refers to the intentional integration of marginalized, underserved, and affected populations, such as people living in poverty, women, displaced persons, and people with disabilities, into all aspects of NTD programming, policymaking, and service delivery. It emphasizes equitable access to healthcare, meaningful participation in decision-making, and the removal of social, economic, and structural barriers that hinder full engagement in prevention, treatment, and support services. Inclusion goes beyond clinical access to encompass empowerment, representation, and the recognition of intersecting forms of exclusion that perpetuate health inequities.

**Figure 4:**
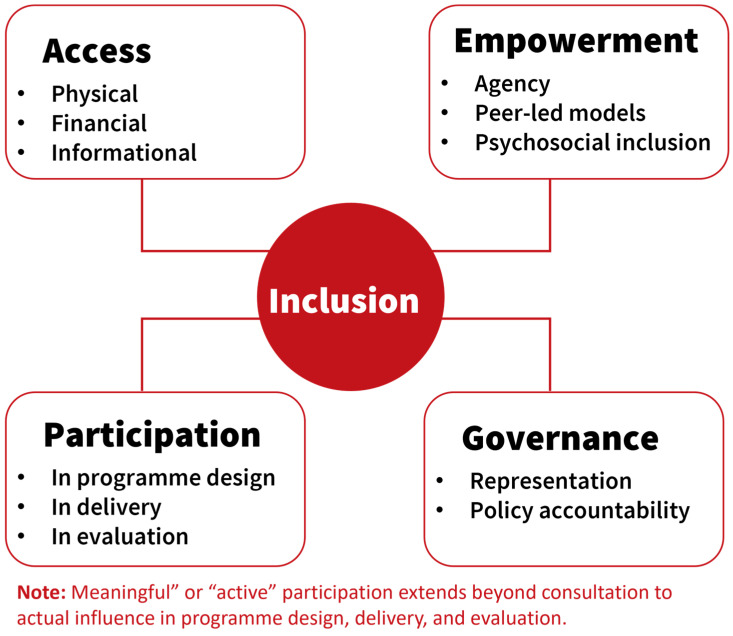
Definition of Inclusion in NTDs developed by CBM based on the findings.

Figure 4 shows how inclusion is interlinked across four domains, access, participation, empowerment, and governance, necessary for building equitable NTD programmes.

This definition aligns with the themes in our review, including the intersection of disability and gender, systemic exclusion, and the role of community empowerment in NTD responses [7,59,60].

Taken together, this review calls for a paradigm shift: from treating NTDs solely as biomedical issues to addressing them as deeply social, structural, and political problems. Inclusion must be embedded across all levels of NTD programming, from community engagement to policy design and implementation, if global health equity goals are to be realised.

### Practical Implementation of Inclusion in NTD Programming

Operationalising inclusion in NTD programming can be facilitated practically through initiatives such as the NTD Inclusion Score Card (NISC), which serves as a tool for NTD organisations to assess and enhance their inclusivity practices by identifying gaps and fostering accountability, aiming to improve the effectiveness and relevance of NTD interventions [77,78].

Additionally, research has highlighted the importance of community-based groups (CBGs) and initiatives, as well as centring OPDs and persons affected in all stages of programming to support inclusion. In their paper, Hotopf et al. map out evidence on existing CBG models for skin-NTDs and lay out a best-practice framework across domains such as self-care, mental health, livelihoods, governance, and advocacy. This model has been designed to include people affected by NTDs in planning, delivery, and evaluation of services, embedding inclusion into the programme [79].

The NISC, on the one hand, is a tool for measuring and improving inclusion in NTD organisations, while the community-based models presented by Hotopf et al. demonstrate how inclusion can be implemented on the ground [78,79].

Similarly, Zongo et al. argue that despite advances in NTD control and elimination programmes, many affected communities remain excluded due to social, economic, and geographic barriers. They explore how civil society organisations (CSOs) play a critical role in advancing equity and inclusion in NTD programmes by acting as trusted community intermediaries, advocating for marginalised groups, enhancing accountability, and ensuring that NTD services reach the most underserved and hard-to-reach populations, thereby strengthening the sustainability and impact of global health efforts [68].

Another aspect that should be a focus for inclusion in NTD programmes is data. Kuper (suggests that Disability and morbidity have not been routinely measured in NTD programmes, and this lack of data hinders advocacy efforts, impedes effective planning and implementation of interventions, and limits the ability to monitor, evaluate programme impact, and secure funding. Collecting such data and utilising it supports more effective planning, enhances advocacy and resource mobilisation, and ensures that interventions are inclusive and responsive to the needs of affected populations [7,8].

From these, we can highlight that although there is still a long way to go when it comes to the concept of inclusion in NTD programming, the area is being advanced through tools like the NTD Inclusion Score Card, community-based models, and stronger data-driven practices. These approaches emphasise the involvement of affected persons in planning and service delivery, with civil society organisations playing a crucial role in reaching marginalised groups. Additionally, better data on disability and morbidity are essential for effective planning, advocacy, and inclusive impact.

## 5. Limitations

As a narrative review, the methodology of this paper prioritises depth and thematic synthesis over exhaustive or systematic coverage, which may introduce selection bias. Although rigorous inclusion and exclusion criteria were applied, relevant studies may have been inadvertently omitted due to publication language (English-only) or date restrictions (2010–2023). While narrative reviews provide a broad and flexible overview of a topic, they are subject to several limitations that can affect the validity and reproducibility of their conclusions. One major limitation is the potential for selection bias, as the inclusion of studies is often based on the authors’ subjective judgement rather than a systematic process [80]. This can lead to incomplete coverage of the literature and an overemphasis on particular viewpoints or findings [26]. Additionally, narrative reviews often lack a standardised methodological framework, which can lead to inconsistencies in how evidence is synthesised and interpreted [77].

Secondly, the heterogeneity in the methodological quality and design of the included studies, ranging from qualitative case studies to mixed-methods research, limits direct comparison and generalizability of findings. Another limitation of this study could be the lack of use of quantitative synthesis or risk-of-bias scoring that would be part of different research methods. Additionally, the review primarily draws from studies based in sub-Saharan Africa and Latin America, which, while highly relevant, may not fully reflect regional dynamics in Asia or the Middle East. Finally, the evolving and context-specific nature of “inclusion” itself means that interpretations may vary across disciplines and cultural settings, affecting the consistency of its conceptualization and operationalization across studies.

### Recommendations for Future Research

While this review highlights strategies for embedding inclusion into NTD programming, it also reveals critical gaps that warrant further investigation. Future research should focus on operationalizing inclusion through concrete programmatic interventions and measurable indicators. There is a pressing need for studies that test the effectiveness of inclusive approaches in practice, particularly in health systems, community engagement, and mass drug administration efforts, and how they impact health outcomes for marginalised groups.

Additionally, there is a limited understanding of how intersecting forms of exclusion (e.g., gender, disability, displacement, and socioeconomic status) combine to hinder access to care. Research should employ intersectional frameworks to explore these dynamics more deeply and guide the design of responsive, context-specific interventions. Finally, more evidence is needed from underrepresented regions, such as Asia and the Middle East, as well as on financing models that support equity-driven programming. Strengthening monitoring and evaluation systems with disaggregated data and participatory feedback loops will be key to advancing the inclusive NTD agenda.

## 6. Conclusions

Inclusion is not peripheral but foundational to the success of NTD programmes. This review highlights the multifaceted nature of exclusion rooted in socioeconomic disparities, gender dynamics, displacement, disability, and systemic neglect and demonstrates how such exclusion undermines progress toward disease control and elimination. Yet, the literature also provides a hopeful trajectory: inclusive, community-driven, and gender-responsive interventions show significant promise in increasing coverage, equity, and sustainability. Achieving the targets outlined in the WHO NTD 2030 Roadmap and the SDGs requires reframing inclusion not just as a principle but as a strategic priority. Moving forward, national governments, implementing partners, and donors must embed inclusion into health systems planning, allocate resources for community empowerment, and institutionalise equity-based monitoring and evaluation frameworks. Only by doing so can we ensure that the fight against NTDs leaves no one behind.

## Figures and Tables

**Table 1:** Eligibility Criteria.

Eligibility Criteria	
Inclusion Criteria	Exclusion Criteria
Studies published in English	Studies published in languages other than English
Studies published from 2010–2025	Studies published before 2010
Primary research studies, including quantitative, qualitative, and mixed-methods studies	Review articles, commentaries, and editorials.
Studies focusing on NTDs and the concept of inclusion, covering various dimensions of inclusion such as access to healthcare, social participation, and empowerment. Additionally, other public health interventions and how they look at inclusion to plan their projects was also looked at.	Studies not directly related to NTDs or the concept of inclusion within public health
Studies that provide clear definitions, frameworks, or models related to inclusion in the context of NTDs.	Studies lacking relevant data or clear definitions of inclusion

**Table 2:** Summary of Findings.

Theme	Key Method	Geographic Focus	Population Targeted	Main Findings	Publication Year	Sample Type	Study Count
1. Social and Economic Inequalities	Qualitative, Mixed-Methods, Systematic Review	Brazil, Sub-Saharan Africa	Racial minorities, Poor, Displaced	- Social and economic inequalities, including poverty, racism, gender discrimination, and displacement, significantly increase vulnerability to NTDs and limit access to care, especially among marginalised and underserved populations. - To eliminate NTDs effectively, programmes must adopt inclusive, equity-focused approaches that address structural barriers, ensure representation in data systems, and tailor services to meet the diverse needs of affected communities.	2018–2025	Mixed: affected populations, national data	22
2. Gender Equity	Qualitative, Gender Frameworks (Women’s Empowerment Framework (WEF), Socio Ecological Model (SEM).	Uganda, Ethiopia, Benin	Women (esp. pregnant/lactating), Men	- Gender norms, power dynamics, and lack of sex-disaggregated data shape access. - Gender-related barriers, worsened by factors like poverty and disability, limit women’s access to NTD treatment and reduce programme effectiveness. - Adopting gender-responsive strategies improves equity, increases treatment coverage, and supports progress toward universal health goals.	2011–2023	Women, Men, Programme implementers	11
3. Inclusive Delivery and Intervention	Participatory Action Research, Case Studies	Multi-Country	Migrant workers, Women, Out-of-school youth	- Rigid models miss marginalised groups; inclusive design improves equity - Effective NTD treatment delivery is hindered by weak health systems and lack of community engagement, but participatory, locally driven approaches and strong health-system integration improve coverage, trust, and sustainability. - Empowering communities, integrating NTD services with broader healthcare, and using disaggregated data to identify and reach underserved groups are essential to closing equity gaps and achieving elimination goals.	2021–2024	Community members, CDDs, Health workers	9
4. Health Systems Inclusion	Systematic Review, Policy Case Study	Liberia, Global Review	Women, Children, Persons with Disabilities	- Integrated, equity-driven systems are needed but are underdeveloped - NTD programmes are moving toward integrated, people-centred models embedded in national health systems, which improve coverage and cost-effectiveness when supported by strong governance, stable financing, and community participation. - Achieving sustainable and equitable NTD care requires embedding intersectional, gender-sensitive planning, reducing donor dependence through domestic resource mobilisation, and centring local civil society in micro-planning and accountability.	2019–2025	Health system actors, Policy documents	8
5. Inclusion of Community Members in NTD Programmes	Community-Based Participatory Research (CBPR), Programme Evaluation	Multi-country Africa	Affected persons, Community volunteers	- Peer-led groups and Community Directed Distributors (CDDs) empower communities and improve outcomes - Inclusive community engagement with trained, diverse distributors and peer support boosts treatment adherence and participation for marginalised groups in NTD programmes. - Formalising distributor roles, providing disability-focused training, and using detailed data monitoring are essential to prevent exclusion and improve care equity.	2017–2025	Persons with disabilities, CDDs, Peer groups	10

## Data Availability

No new data were gathered or developed; the findings are based on reviews of currently existing research.

## Abbreviations

The following abbreviations are used in this manuscript:

CDD	Community Directed Distributers
CSO	Civil Society Organization
DMDI	Disease Management, Disability, and Inclusion
MDA	Mass Drug Administration
NTDs	Neglected Tropical Diseases
SDGs	Sustainable Development Goals

## References

[1] World Health Organization (2021). A Road Map for Neglected Tropical Diseases 2021–2030.

[2] Hotez P.J. (2020). What Constitutes a Neglected Tropical Disease?. PLoS Neglected Trop. Dis..

[3] Branda F., Ali A.Y., Ceccarelli G., Albanese M., Binetti E., Giovanetti M., Ciccozzi M., Scarpa F. (2025). Assessing the Burden of Neglected Tropical Diseases in Low-Income Communities: Challenges and Solutions. Viruses.

[4] World Health Organization (2024). Neglected Tropical Diseases—Key Facts.

[5] Reed S.L., McKerrow J.H. (2018). Why Funding for Neglected Tropical Diseases Should Be a Global Priority. Clin. Infect. Dis..

[6] Houweling T.A., Karim-Kos H.E., Kulik M.C., Stolk W.A., Haagsma J.A., Lenk E.J., Richardus J.H., de Vlas S.J. (2016). Socioeconomic Inequalities in Neglected Tropical Diseases: A Systematic Review. PLoS Neglected Trop. Dis..

[7] Kuper H. (2019). Neglected tropical diseases and disability-what is the link?. Trans. R. Soc. Trop. Med. Hyg..

[8] Kuper H. (2020). Disability, mental health, stigma and discrimination, and neglected tropical diseases. Trans. R. Soc. Trop. Med. Hyg..

[9] Koschorke M., Al-Haboubi Y.H., Tseng P.-C., Semrau M., Eaton J. (2022). Mental health, stigma, and neglected tropical diseases: A review and systematic mapping of the evidence. Front. Trop. Dis..

[10] Kollmann K.M., Abrahamsson S., Jesudason T. (2020). Why disability inclusion is essential for trachoma elimination. Community Eye Health J..

[11] Ochola E.A., Karanja D.M.S., Elliott S.J. (2021). The impact of Neglected Tropical Diseases (NTDs) on health and wellbeing in sub-Saharan Africa (SSA): A case study of Kenya. PLoS Neglected Trop. Dis..

[12] World Health Organization (2022). Global Report on Health Equity for Persons with Disabilities.

[13] Dean L., Ozano K., Adekeye O., Dixon R., Fung E.G., Gyapong M., Isiyaku S., Kollie K., Kukula V., Lar L. (2019). Neglected Tropical Diseases as a ‘litmus test’ for Universal Health Coverage? Understanding who is left behind and why in Mass Drug Administration: Lessons from four country contexts. PLoS Neglected Trop. Dis..

[14] Braveman P., Gruskin S. (2003). Defining equity in health. J. Epidemiol. Community Health.

[15] Cornwall A. (2008). Unpacking ‘Participation’: Models, meanings and practices. Community Dev. J..

[16] World Health Organization (2023). Disability and Health Fact Sheet.

[17] Sun N., Amon J.J. (2018). Addressing Inequity: Neglected Tropical Diseases and Human Rights. Health Hum. Rights.

[18] Trachoma Coalition (2020). Reaching the Furthest Behind First: Disability Inclusion Essential to Universal Trachoma Programmes.

[19] CBM Global (2022). Mental Health of People with NTDs: Towards a Person-Centred Approach.

[20] United Nations (2019). Leave No One Behind: Inclusion of Persons with Disabilities in the 2030 Agenda for Sustainable Development.

[21] World Bank (2024). Disability Inclusion Overview.

[22] United Nations Enable (2021). Factsheet on Persons with Disabilities.

[23] UN Sustainable Development Group (2016). Leave no One Behind: A UN Framework for the 2030 Agenda.

[24] Sukhera J. (2022). Narrative Reviews: Flexible, Rigorous, and Practical. J. Grad. Med. Educ..

[25] Kaushik P., Pati P.K., Khan M.L., Khare P.K. (2022). Flow Diagram for Systematic Literature Review Using SALSA Framework. Environ. Sci. Policy.

[26] Greenhalgh T., Thorne S., Malterud K. (2018). Time to challenge the spurious hierarchy of systematic over narrative reviews?. Eur. J. Clin. Investig..

[27] Ferrari R. (2015). Writing narrative-style literature reviews. Med. Writ..

[28] da Conceição J.R., Lopes C.P.G., Ferreira E.I., Epiphanio S., Giarolla J. (2022). Neglected Tropical Diseases and Systemic Racism, Especially in Brazil: From Socio-Economic Aspects to the Development of New Drugs. Acta Trop..

[29] Simionato de Assis I., Arcoverde M.A.M., Ramos A.C.V., Alves L.S., Berra T.Z., Arroyo L.H., Queiroz A.A.R.D., Santos D.T.D., Belchior A.D.S., Alves J.D. (2018). Social Determinants, Their Relationship with Leprosy Risk and Temporal Trends in a Tri-Border Region in Latin America. PLoS Neglected Trop. Dis..

[30] Dyal J.W., Grant M.P., Broadwater K., Bjork A., Waltenburg M.A., Gibbins J.D., Hale C., Silver M., Fischer M., Steinberg J. (2020). COVID-19 Among Workers in Meat and Poultry Processing Facilities—19 States, April 2020. MMWR Morb. Mortal. Wkly. Rep..

[31] Mackey T.K., Ayers C.K., Kondo K.K., Saha S., Advani S.M., Young S.D., Spencer H., Rusek M., Anderson J., Veazie S. (2021). Racial and Ethnic Disparities in COVID-19-Related Infections, Hospitalizations, and Deaths: A Systematic Review. Ann. Intern. Med..

[32] Minority Rights Group, Vidas Negras com Deficiência Importam (2022). The Situation of Black People with Disabilities in Brazil.

[33] Polack S., Delgado Ramos V., Sepúlveda Köptcke L., de Araujo Morais I., Reichenberger V., Scherer N., Veloso de Albuquerque M.D.S., Kuper H., Lyra T.M., Moran de Brito C.M. (2025). Disability Inclusion in the Brazilian Health System: Results of a Health System Assessment. Glob. Health Action.

[34] Clark J., Davis E.L., Prada J.M., Gass K., Krentel A., Hollingsworth T.D. (2023). How Correlations Between Treatment Access and Surveillance Inclusion Impact Neglected Tropical Disease Monitoring and Evaluation—A Simulated Study. PLoS Neglected Trop. Dis..

[35] Magalhães A.R., Codeço C.T., Svenning J.-C., Escobar L.E., Van de Vuurst P., Gonçalves-Souza T. (2023). Neglected Tropical Diseases Risk Correlates with Poverty and Early Ecosystem Destruction. Infect. Dis. Poverty.

[36] Houweling T.A.J., Kristensen T.K., Dada N., D’Amico G. (2016). Socio-Economic Inequality in Neglected Tropical Disease Risk. Trans. R. Soc. Trop. Med. Hyg..

[37] United Nations High Commissioner for Refugees (2019). Working with Persons with Disabilities in Forced Displacement.

[38] UNHCR (2022). Guidance: Identification of Persons with Disabilities at Registration Using the Washington Group Questions.

[39] Elmukashfi ShamsEldin Elobied H., Bushra Masaad Ahmed M., Balla Ahmed A.M., Abdelrahman Hassan Salih R., Mohammed Mokhtar Ahmed S., Mahgoub A.M.A., Hashim Abdelmagid Mohamed A., Hamid Abdallah Elamin E., Osman Khalafalla El-Haj A.-R.M., Hamed Abd-Allah M.A.-H. (2025). Healthcare Accessibility, Utilisation and Quality of Life among Internally Displaced Persons in Somalia: A Cross-Sectional Study. Confl. Health.

[40] Kelley-Hope L., Harding-Esch E.M., Willems J., Ahmed F., Sanders A.M. (2023). Conflict–Climate–Displacement: A Cross-Sectional Ecological Study Determining the Burden, Risk and Need for Strategies for Neglected Tropical Disease Programmes in Africa. BMJ Open.

[41] Harvey D., Shu’aibu J., Debam M.T., Aba A.K., Torres-Vitolas C.A. (2022). How can the neglected tropical disease community be inclusive and equitable in programme delivery? Reaching refugees and internally displaced persons through integrating a ‘leave no one behind’ approach. Int. Health.

[42] Adams M.W., Sutherland E.G., Eckert E.L., Saalim K., Reithinger R. (2022). Leaving no one behind: Targeting mobile and migrant populations with health interventions for disease elimination—A descriptive systematic review. BMC Med..

[43] International Disability Alliance (2023). Advancing Disability-Inclusive Strategies at the Global Refugee Forum.

[44] Women’s Refugee Commission, UNHCR (2020). Translating Disability-Inclusive Policy into Practice in Humanitarian Settings.

[45] Rust J., Clark A., Woodgate M., Koch C., Mohammed T., Steinmann P., Krentel A., Torres-Vitolas C.A., Carlin A., Pavluck A. (2022). Innovate to eliminate: A prerequisite in NTD programmes. Int. Health.

[46] Elrha (2023). Investing in Inclusive WASH: Examining Barriers and Value in Humanitarian Response.

[47] United Nations Children’s Fund (UNICEF) (2022). Including Children with Disabilities in Humanitarian Action: WASH.

[48] Sangare M., Diabate A.F., Coulibaly Y.I., Tanapo D., Thera S.O., Dolo H., Dicko I., Coulibaly O., Sall B., Traore F. (2024). Understanding the barriers and facilitators related to never treatment during mass drug administration among mobile and migrant populations in Mali: A qualitative exploratory study. BMJ Glob. Health.

[49] Obani P. (2023). Inclusiveness in Humanitarian Action: Access to Water, Sanitation and Hygiene for People with Disabilities. Int. J. Disaster Risk Reduct..

[50] Theobald S., MacPherson E.E., Dean L., Jacobson J., Ducker C., Gyapong M., Hawkins K., Elphick-Pooley T., MacKenzie C., Kelly-Hope L. (2017). 20 years of gender mainstreaming in health: Lessons and reflections for the neglected tropical diseases community. BMJ Glob. Health.

[51] Cohn D.A., Kelly M.P., Bhandari K., Zoerhoff K.L., Batcho W.E., Drabo F., Negussu N., Marfo B., Goepogui A., Lemoine J.F. (2019). Gender equity in mass drug administration for neglected tropical diseases: Data from 16 countries. Int. Health.

[52] Rilkoff H., Tukahebwa E.M., Fleming F.M., Leslie J., Cole D.C. (2013). Exploring Gender Dimensions of Treatment Programmes for Neglected Tropical Diseases in Uganda. PLoS Neglected Trop. Dis..

[53] Mushi V. (2021). Implementation challenges of community directed treatment with ivermectin program for control of onchocerciasis in Ulanga, Tanzania. East Afr. Health Res. J..

[54] University of Washington START Center (2021). Neglected Tropical Diseases: Women and Girls in Focus.

[55] World Health Organization (2011). World Bank. World Report on Disability.

[56] Wharton-Smith A., Rassi C., Batisso E., Ortu G., King R., Endriyas M., Counihan H., Hamade P., Getachew D. (2019). Gender-Related Factors Affecting Health Seeking for Neglected Tropical Diseases: Findings from a Qualitative Study in Ethiopia. PLoS Neglected Trop. Dis..

[57] Geyer R.E., Ibikounlé M., Emmanuel-Fabula M., Roll A., Avokpaho E., Elijan A., Wèkè L.C., Togbevi C.I., Chabi F., Houngbégnon P. (2020). Gender norms and mass deworming program access in Comé, Benin: A qualitative assessment of gender-associated opportunities and challenges to achieving high mass drug administration coverage. PLOS Neglected Trop. Dis..

[58] Arney J.K., Headland M.K., Bertone A.M., Meite A., Ettiegne-Traore V., Asemanyi-Mensah K., Dede Teiko Dzathor I., Kargbo-Labour I., Jalloh U., Houck P. (2023). Synthesis of findings from the literature and a qualitative research study on the impacts of gender, disability, and ethnicity in Neglected Tropical Diseases programs. PLoS Neglected Trop. Dis..

[59] George N.S., David S.C., Nabiryo M., Sunday B.A., Olanrewaju O.F., Yangaza Y., Shomuyiwa D.O. (2023). Addressing neglected tropical diseases in Africa: A health equity perspective. Glob. Health Res. Policy.

[60] Ochola E., Karanja S., Elliott S. (2022). Local tips, global impact: Community-driven measures as avenues of inclusion for NTD control in western Kenya. Infect. Dis. Poverty.

[61] Piotrowski H., Gwani N., Yashiyi J., Oluwole A., Ayuba S., Surakat M., Adekeye O., Lar L., Kevin D.G., Lawong B.D. (2023). Promoting equity through inclusive learning, planning and implementation of mass drug administration in Nigeria. Int. Health.

[62] Dean L., Tolhurst R., Nallo G., Kollie K., Bettee A., Theobald S. (2023). A health-systems journey towards more people-centred care: Lessons from neglected tropical disease programme integration in Liberia. Health Policy Plan..

[63] World Health Organization (2024). Collaboration for the Control of Neglected Tropical Diseases. https://www.who.int/teams/control-of-neglected-tropical-diseases/collaboration.

[64] Amazigo U., Leak S.G., Zoure H.G., Okoronkwo C., Diop Ly M., Isiyaku S., Crump A., Okeibunor J.C., Boatin B. (2021). Community-directed distributors—The “foot soldiers” in the fight to end NTDs. PLoS Neglected Trop. Dis..

[65] Banda G.T., Deribe K., Davey G. (2021). How can we better integrate the prevention, treatment, control and elimination of neglected tropical diseases with other health interventions? A systematic review. BMJ Glob. Health.

[66] World Health Organization (2024). Ending NTDs Together Towards 2030: Progress Report.

[67] Research for Development (R4D) (2022). Mobilising Domestic Financing for Neglected Tropical Diseases: Five Lessons for Policy-Makers.

[68] Zongo K., Mberu M., Assefa L., Kucera T., Pappa S., Amin A., Sankar G. (2025). Bridging the last mile: The critical role of local civil society in advancing equity and inclusion for neglected tropical diseases. Int. J. Infect. Dis..

[69] Alderton D.L., Ackley C., Trueba M.L. (2024). The Psychosocial Impact of Skin-Neglected Tropical Diseases: A Scoping Review. Front. Trop. Dis..

[70] CBM Global Disability Inclusion (2023). Community-Based Peer Groups and Psychosocial Support in Humanitarian Settings.

[71] Seekles M.L., Kadima J.K., Ding Y., Bulambo C.B., Kim J.J., Kukola J.K., Omumbu P.O., Mulamba R.M., Nganda M., Ngenyibungi S.M. (2023). Mental health, stigma and the quality of life of people affected by neglected tropical diseases of the skin in Kasai Province, Democratic Republic of the Congo: A sex-disaggregated analysis. Int. Health.

[72] Nayak P.K., Mackenzie C.D., Agarwal A., van Wijk R., Mol M.M., Eaton J., Semrau M., Valle-Trabadelo C., Kaloiya G.S., Pratap A. (2025). A new guide for basic psychological support for persons affected by neglected tropical diseases: A peer support tool suitable for persons with a diagnosis of leprosy and lymphatic filariasis. PLOS Neglected Trop. Dis..

[73] Badu E., Murphy R., Agyemang D., Jolley E. (2018). Ghana Disability Data Disaggregation Pilot Project: Results of Integrating Disability into Routine Data Collection Systems.

[74] Krentel A., Gyapong M., Mallya S., Boadu N.Y., Amuyunzu-Nyamongo M., Stephens M., McFarland D.A. (2017). Review of the factors influencing the motivation of community drug distributors towards the control and elimination of neglected tropical diseases (NTDs). PLoS Neglected Trop. Dis..

[75] Worrell C.M., Brant T.A., Javel A., Denis E., Fayette C., Monestime F., Knowles E., Bennett C., Utzinger J., Odermatt P. (2025). Microplanning improves stakeholders’ perceived capacity and engagement to implement lymphatic filariasis mass drug administration. PLoS Neglected Trop. Dis..

[76] The Guardian (2025). ‘Some of These Diseases Are in the Bible’: Despair as Cuts Halt Progress on Neglected Tropical Diseases. https://www.theguardian.com/global-development/2025/apr/09/despair-as-cuts-halt-progress-on-neglected-tropical-diseases-usaid.

[77] Baethge C., Goldbeck-Wood S., Mert95ens S. (2019). SANRA—A scale for the quality assessment of narrative review articles. Res. Integr. Peer Rev..

[78] van Wijk R., Baudoin S.J.M., Ejiogu B., Regmi U., Duck M., Rabiu I., Vettel C., Broekkamp H., Geutjes R., Peters R.M.H. (2025). From words to action: The development of the Neglected Tropical Disease Inclusion Score Card (NISC). Infect. Dis. Poverty.

[79] Hotopf I., Chowdhury S., Robert G., Sellers M.E., Phillip M., Fastenau A., del Mar Marais M., Vettel C., Rahman M., Singh R.K. (2025). Community-based models for neglected tropical diseases affecting the skin: A scoping review. Front. Trop. Dis..

[80] Grant M.J., Booth A. (2009). A typology of reviews: An analysis of 14 review types and associated methodologies. Health Inf. Libr. J..

